# Inflammatory and Metabolic Blood Parameters Associated with Aggression, Impulsivity, and Suicide Risk Among Male Patients with Antisocial Personality Disorder in a Forensic Psychiatry Unit in Turkey: A Cross-Sectional Comparative Study

**DOI:** 10.3390/diagnostics16060831

**Published:** 2026-03-11

**Authors:** Berçem Afşar Karatepe, Gülay Tasci

**Affiliations:** 1 Internal Medicine Department, Elazığ Fethi Sekin City Hospital, Elazığ 23100, Türkiye; 2 Mental Health and Disorders Department, Elazığ Fethi Sekin City Hospital, Elazığ 23100, Türkiye

**Keywords:** antisocial personality disorder, forensic psychiatry, inflammation, metabolic parameters, aggression and impulsivity, suicide risk

## Abstract

**Background/Objectives:** Antisocial personality disorder (ASPD) is strongly associated with violence, substance use, criminal behavior, and elevated suicide risk. Although inflammatory and metabolic dysregulation have been implicated in severe psychiatric disorders, the biological correlates of impulsivity, aggression, and suicide risk in forensic ASPD populations remain unclear. This study aimed to investigate whether routine hematological, inflammatory, and metabolic parameters are associated with these clinical features. **Methods:** This cross-sectional study included 57 male individuals diagnosed with antisocial personality disorder (ASPD) who had committed crimes and were referred to the Forensic Psychiatry Department of Elazığ Fethi Sekin City Hospital in Turkey by the court, and 56 age-matched healthy controls. Participants completed standardized assessments of impulsivity (BIS-11), aggression (BPAQ), and suicide probability (SPS). Hematological indices, inflammatory markers, and routine biochemical parameters were analyzed. Group comparisons, correlation analyses, and multivariable logistic regression were performed. **Results:** Compared with age-matched controls, individuals with ASPD showed markedly higher impulsivity, aggression, and suicide probability, alongside substantially higher rates of substance use, imprisonment history, and suicide attempts (all *p* < 0.001). Hematological and inflammatory analyses revealed lower red blood cell (RBC) counts and elevated mean corpuscular volume (MCV), red cell distribution width (RDW), C-reactive protein (CRP), neutrophil-to-lymphocyte ratio (NLR), and CRP–albumin ratio (CAR) in the ASPD group (all *p* < 0.05). Biochemical profiling showed reduced glucose, total protein, albumin, HDL, ALT, and vitamin B12 levels, with increased uric acid levels in ASPD (*p* < 0.05). Multivariable analysis indicated that being married and having higher education were protective against ASPD, whereas higher uric acid and CAR levels were associated with increased risk. **Conclusions:** The findings indicate that criminal offenders with ASPD show increased inflammatory markers and altered hematological and biochemical profiles. Routine blood parameters, combined with psychometric assessments, may help identify individuals at higher behavioral risk and support early risk stratification in forensic psychiatric settings, although causal relationships cannot be inferred from this cross-sectional study.

## 1. Introduction

Antisocial personality disorder (ASPD) is a personality disorder pattern characterized by key features such as a persistent disregard for the rights of others, deceptive behavior, refusal to comply with social and legal laws, impulsive behavior, aggressive tendencies, dangerous actions, and committing numerous crimes. According to DSM-5, ‘conduct disorder’ that emerges in childhood/adolescence is a precursor to antisocial personality disorder [1]. ASPD appears at higher rates among people who receive forensic psychiatric treatment because they show repeated criminal behavior and substance abuse and alcohol problems, and they face a high risk of suicide [2,3]. The behavioral and functional features of ASPD create significant challenges for affected individuals. Due to the ego-syntonic nature of the disorder, engagement in treatment is often limited. Research evidence suggests that ASPD develops through a combination of genetic predisposition, neurobiological alterations, and environmental influences [4].

The neural systems that control behaviors maintain direct connections with stress response mechanisms and reward processing systems, which indicates that ongoing biological stress could lead to harmful behavioral patterns in ASPD [2]. Aggression, impulsivity, and suicidal tendencies are not independent concepts. These three behavioral patterns are overlapping manifestations of impaired behavioral inhibition and emotional dysregulation. These dimensions share common neurobiological underpinnings, particularly in the prefrontal–limbic circuits that regulate impulse control and emotional processing, involving serotonergic dysfunction, inflammatory activation, and metabolic dysregulation. In individuals with antisocial personality disorder, deficits in executive control and heightened responses to stress can predispose to both extroverted (aggression and violence) behaviors and introverted (suicidal acts) behaviors [5]. The data in the literature support the relationship between suicidal tendencies and aggression, especially anger related to personality traits. It has been reported that anger, which increases with personality traits, increases suicidal tendencies and violence, which are linked to emotion regulation disorders and impulsivity [6]. Therefore, considering these areas together can provide a more comprehensive understanding of the common biological mechanisms underlying risky behaviors in forensic populations.

Research into psychiatric biomarkers today investigates both standard inflammatory markers and standard blood tests that check hematological and biochemical values [7]. The SII (serum inflammation index), together with NLR, CRP, and PLR (platelet–lymphocyte ratio), shows evidence that these biomarkers relate to symptom severity, treatment response, and suicidal behavior [8,9,10]. Studies from the last few years demonstrate that suicide attempters have abnormally low blood albumin levels, and their neutrophil-to-albumin ratio (NAR) increases [11]. Research shows that inflammatory cytokines affect both monoaminergic neurotransmission and neuroplasticity, so scientists now believe that inflammation can cause people to develop more impulsive behavior and aggressive tendencies [12]. Some studies have linked low blood albumin and vitamin B12 levels to depression, cognitive decline, and an increased risk of suicide [13,14]. There are also studies reporting a link between changes in lipid profiles and an increased risk of suicide [15]. Research findings on this topic show no agreement across studies, and there is little scientific evidence about blood parameters in ASPD cases that involve forensic investigation [16,17].

There is no current research that studies aggression together with impulsivity, suicide risk, and criminal conduct and their relationship to blood tests and chemical, hormonal, and inflammatory markers in people who have ASPD. The combination of psychometric instruments with biological markers could establish an improved risk assessment system that doctors could use to create individualized treatment plans for their patients in forensic settings. Therefore, the main aim of our article is to elucidate the biological mechanisms behind behavioral patterns such as aggression, impulsivity, and suicidal behavior in individuals with ASPD who have committed crimes.

## 2. Materials and Methods

### 2.1. Ethics Committee Approval

The research was performed in compliance with the Declaration of Helsinki. Approval was obtained from the Fırat University Non-Interventional Local Ethics Committee, bearing project number 2022/02-56, after its meeting on 10 February 2022.

### 2.2. Criteria for Inclusion and Exclusion

Many psychiatric disorders affecting criminal responsibility, such as psychotic disorders, bipolar disorder, substance use disorders, major depression, and ASPD (Abnormal Psychological Disorder) cases, are referred to forensic psychiatry services by courts. This study included 57 male individuals aged 18–65 years who were referred to the Forensic Psychiatry Service of Elazığ Fethi Sekin City Hospital by courts for forensic observation/evaluation between 15 June 2022, and 15 June 2023, and were diagnosed with antisocial personality disorder (ASPD). The ASPD diagnosis was made according to DSM-5 diagnostic criteria based on a clinical interview conducted by a psychiatrist. In accordance with court orders, the stay in the forensic psychiatry service is limited to a maximum of three weeks. Individuals with intellectual disabilities, individuals with limited literacy, or those already diagnosed with other mental disorders were excluded. Individuals with chronic illnesses, a history of diagnosed cancer, poor general health status, renal or hepatic dysfunction, or any medical condition associated with systemic or localized edema or inflammation were excluded from the study. A healthy control group consisted of 56 male individuals with similar age, gender, and body mass index who did not have a diagnosis of ASPD and/or any other psychiatric diagnosis. The same medical exclusion criteria were applied to the healthy control group. Whether the healthy control group had a psychiatric or medical condition was assessed by a qualified psychiatrist, and their past medical records were reviewed. Participants provided written and verbal informed consent. A trained mental health professional gathered background information before handing over standard questionnaires. All participants completed a sociodemographic data form, the Buss–Perry Aggression Questionnaire (BPAQ), the Barratt Impulsiveness Scale-11 (BIS-11), and the Suicide Probability Scale (SPS). The study used scales that were in Turkish and had proven validity and reliability in the Turkish population. Blood samples were taken from patients on their first day of hospitalization while they were fasting.

### 2.3. Data Collection Tools

Sociodemographic and clinical data form: A semi-structured demographic and clinical information form prepared by us was administered to determine the participants’ sociodemographic characteristics such as marital status, age, educational level, alcohol–substance use, and suicide attempt. The form also included items related to clinical and legal history, such as previous interactions with the legal system, incarceration history, and the nature of the crime leading to imprisonment.

Buss–Perry Aggression Questionnaire (BPAQ): A Likert-type measure that examines aggression in four dimensions: physical aggression, anger, hostility, and verbal aggression. Responses were recorded on a scale ranging from 1 (“never or almost never applies to me”) to 5 (“applies to me very often”). In this study, internal consistency coefficients were satisfactory for all subscales (physical aggression α = 0.82; verbal aggression α = 0.75; hostility α = 0.80; anger α = 0.85) and were largely consistent with those reported in the original scale development study [18]. The Turkish adaptation, including validity and reliability analyses, had previously been conducted by Madran et al. [19].

Barratt Impulsiveness Scale-11 (BIS-11): A widely used self-assessment tool designed to measure impulsive personality traits. The scale consists of three main dimensions: attentional impulsivity (reflecting difficulties in sustained attention and cognitive organization), motor impulsivity (reflecting behavioral impulsivity and impatience), and planning impulsivity (reflecting lack of future orientation and cognitive control). Subscale scores and total impulsivity scores were calculated; higher scores indicate greater impulsivity. The psychometric properties of the Turkish version of the BIS-11 were determined by Güleç et al. [20].

Suicide Probability Scale (SPS): A 36-item self-assessment tool developed to estimate the likelihood of suicidal behavior in adolescent and adult populations. Items are rated using the SPS, a Likert-type scale, and the total score represents the overall level of suicide risk; higher scores indicate increased risk [21]. The Turkish adaptation of the scale and its initial validity assessment were first conducted by Tuğcu, followed by reliability and validity analyses in clinical samples. Previous studies have reported high internal consistency (α = 0.87), excellent test–retest reliability (0.98), and strong convergent validity (0.84) for the total score, supporting the scale’s robustness in Turkish populations [22].

### 2.4. Laboratory Samples

Venous blood samples were obtained from the antecubital vein of all participants after an average fasting period of 12 h and collected into EDTA-containing tubes. The following hematological parameters were recorded: total white blood cell count, hemoglobin, platelet count, hematocrit, mean platelet volume (MPV), differential leukocyte counts (neutrophils, eosinophils, lymphocytes, monocytes, and basophils), and red cell distribution width (RDW). Based on these measurements, neutrophil-to-lymphocyte ratio (NLR), platelet-to-lymphocyte ratio (PLR), monocyte-to-lymphocyte ratio (MLR), neutrophil-to-albumin ratio (NAR), CRP-to-albumin ratio (CAR), and the systemic immune-inflammation index (SII) were calculated using standard formulas. Complete blood counts were performed using a Beckman Coulter LH 750 analyzer (Beckman Coulter, Inc., Brea, CA, USA) based on the impedance principle. Serum albumin concentrations were measured by a spectrophotometric method using the Cobas 8000 series c702 modular analyzer (Roche Diagnostics, Rotkreuz, Switzerland). Measurements of fasting blood glucose, fasting insulin, total cholesterol, triglycerides, HDL-cholesterol, LDL-cholesterol, alanine aminotransferase, aspartate aminotransferase, blood urea nitrogen, and creatinine were carried out using enzymatic colorimetric assays on an automated Mindray (China) analyzer (Mindray, Shenzhen, China). C-reactive protein (CRP) levels were determined with reagents from Wako Chemicals (Osaka, Japan) on a Hitachi 7600 chemistry analyzer.

### 2.5. Statistical Analysis

With SPSS 21.0, statistical checks followed standard procedures. Histograms paired with the Kolmogorov–Smirnov method helped assess how continuous data spread across ranges. Categorical types outlined count totals and share values instead. When looking at measurement types that are numerical, differences across categories were checked via *t*-tests or ANOVA if distribution seemed regular. When numbers failed to follow a regular pattern, scientists turned to the Mann–Whitney U method or the Kruskal–Wallis approach. In suitable cases, they applied Fisher’s Exact or Chi-square analysis on groups. To measure how variables are linked together, they reached for Pearson or Spearman scores to reflect association strength. When results fall below 0.05, researchers often consider them statistically meaningful. From the analysis, certain parameters stood out—showing clear distinctions between healthy individuals and those with antisocial personality disorder in single-variable checks. These differences then moved into a logistic regression framework for further exploration. Before combining variables, checks were made to avoid strong correlations among candidates. On top of that, a multivariable logistic regression—using a backward stepwise method—was run. What stood out were the variables still significant (*p* < 0.05) after everything was accounted for; those counted as independent predictors for antisocial personality disorder. To check how well the model fit real data, the Hosmer–Lemeshow test helped compare observed versus predicted outcomes. For every predictor, odds ratios and a range of 95% confidence were reported to show their effects.

## 3. Results

The study included a total of 113 male participants: 57 individuals diagnosed with ASPD and 56 healthy controls. There was no statistically significant difference in age between the ASPD group (32.37 ± 6.61 years) and the control group (33.04 ± 8.45 years) (*p* > 0.05). There was no significant difference between the two groups in terms of body mass index (*p* = 0.376). The ASPD group members showed lower socioeconomic status, educational attainment, and employment rates than the control group participants at a significance level of *p* < 0.001. The ASPD group showed significantly higher rates of alcohol consumption, substance abuse, history of imprisonment, self-injurious behavior, and suicidal thoughts (54.4%, 75.4%, 80.7%, and 59.6%, respectively; all *p* < 0.001). The ASPD group members committed their crimes mostly through multiple offenses (31.6%), drug-related offenses (17.5%), theft (15.8%) and homicide (8.8%). The study includes detailed information on the sociodemographic factors and clinical data of the participants, which appear in Table 1.

The research found that the ASPD group showed significant differences from control participants in their scores on impulsivity, aggression, and suicide risk assessments (all *p* < 0.001). The BIS-11 results showed that ASPD participants showed significantly elevated scores for the motor impulsivity and non-planning (disorganization) subscales and total impulsivity compared to controls, whereas controls showed elevated attention-related impulsivity scores (*p* < 0.001). All subscale scores and the total score of the BPAQ were significantly elevated in the ASPD group relative to the control group (*p* < 0.001). The SPS showed that people with ASPD had higher scores in all assessment categories, including suicidal thoughts, hopelessness, negative self-perception, hostility, and total test results (*p* < 0.001). Scale score distributions are summarized in Table 2.

The study of hematological parameters revealed that red blood cell measurements between different groups showed major variations. The ASPD group showed lower red blood cell (RBC) counts, larger red blood cell size (higher MCV), and greater size variation (higher RDW) than the control group (all *p* < 0.001). The analysis revealed no statistically meaningful variations between the study groups for hemoglobin (HGB) levels, white blood cell count (WBC), hematocrit (HCT), platelet count (PLT), mean platelet volume (MPV), or leukocyte distribution patterns (neutrophils, lymphocytes, monocytes, eosinophils and basophils) (all *p* > 0.05).

Biochemical and hormonal tests showed that the ASPD group had lower glucose, urea, total protein, albumin, HDL, and vitamin B12 levels than the control group (*p* < 0.05 for all). The ASPD group showed elevated uric acid levels, which reached statistical significance at *p* = 0.019. The ASPD group also showed lower ALT levels, which reached statistical significance at *p* = 0.001. The research findings showed no differences between the groups regarding creatinine levels, AST results, total cholesterol readings, LDL levels, triglyceride measurements, sodium and potassium electrolyte levels, and TSH, T3, and T4 levels.

The neutrophil-to-lymphocyte ratio (NLR),C-reactive protein (CRP), and C-reactive protein–albumin ratio (CAR) showed significant elevation in the ASPD group compared to the control group (*p* = 0.019, *p* = 0.031, and *p* = 0.005, respectively). The research findings indicated that no statistically meaningful differences existed between the study groups for MLR, PLR, SII, or NAR values. The laboratory results, which include all test results, appear in Table 3.

Correlation analyses within the ASPD group revealed no significant associations between most laboratory parameters and psychometric scale scores. The study discovered that total impulsivity scores showed a small positive connection with basophil levels (r = 0.272, *p* = 0.041), and suicide probability scores showed a small positive connection with ALT levels (r = 0.279, *p* = 0.036). The research found no relationship between aggression scores and any laboratory measurements. The research results appear in Table 4.

No statistically significant relationships were found between crime types and psychometric scale scores or inflammatory markers. The results showed that CRP and CAR values were higher in cases of intentional homicide, but the differences between these values and those from other crime categories did not achieve statistical significance. Correlation results related to crime categories are presented in Table 5.

In the multivariable logistic regression analysis, marital status, education level, uric acid, and CAR were independently associated with antisocial personality disorder. Being married was associated with significantly lower odds of the outcome (OR = 0.17, 95% CI: 0.06–0.52, *p* = 0.002). Higher education level was also an independent protective factor (OR = 0.05, 95% CI: 0.01–0.18, *p* < 0.001). Serum uric acid was positively associated with the outcome; each one-unit increase was associated with a 1.59-fold increase in odds (OR = 1.59, 95% CI: 1.08–2.36, *p* = 0.020). Importantly, each 0.1-unit increase in CAR was associated with a 3.4-fold increase in the odds of the outcome (OR = 3.36, 95% CI: 1.18–7.61, *p* = 0.023). Alcohol use was excluded from the multivariable analysis due to the absence of alcohol consumption in the control group. The results of the regression analysis appear in Table 6.

## 4. Discussion

Patients diagnosed with ASPD in forensic psychiatry had high rates of alcohol and substance use, history of suicide attempts, imprisonment, self-harm behaviors such as cuts and razor marks, and tattoos. High levels of aggression, impulsivity, and suicide risk were observed in this population. Hematological analysis revealed an increase in MCV and RDW, as well as a decrease in RBC count. Biochemical assessment showed decreases in glucose, albumin, total protein, urea, ALT, and vitamin B12 levels, and increases in uric acid concentrations. Inflammatory markers such as CRP, CAR, and NLR were also elevated. Significant correlations were found between impulsivity and suicide risk scales and certain blood parameters. Although no statistically significant relationship was observed between types of criminal behavior and psychometric scales or hematological parameters, a positive correlation was found between CRP and CAR levels in individuals who had committed murder. In multivariable logistic regression analysis, marital status, education level, uric acid, and CAR were independently associated with the antisocial personality disorder.

The findings of this study supported earlier studies showing that people with ASPD diagnosis have lower social economic status than individuals without mental health issues. Research shows that ASPD patients experience economic poverty, unstable marriages, unemployment, and encounter different types of social discrimination, which scientists identify as core environmental factors of the disorder [23,24]. The socioeconomic patterns show how individuals with ASPD must deal with persistent difficulties in building lasting social relationships, keeping jobs, and achieving financial security because of their core behavioral traits. However, it is difficult to assess whether low socioeconomic status and marital status characteristics are the cause or consequence of criminal behavior. The literature tends to address this relationship at the level of association [2,23].

The types of crimes committed by individuals with ASPD are mostly organized/planned and/or profit-driven crimes such as theft, drug use/trafficking, and fraud. Violent crimes such as intentional injury, assault, and murder are also frequently observed, and it has been reported that individuals with ASPD do not show remorse after committing these acts. It has also been reported that individuals diagnosed with ASPD commit crimes repeatedly [25,26]. In individuals diagnosed with schizophrenia and bipolar disorder, it has been reported that violent crimes are committed, mostly directed at family members, and that they develop suddenly during the acute phase of the illness [27]. In our study, consistent with the literature, it was found that individuals diagnosed with ASPD committed multiple crimes, including murder, theft, and drug use/trafficking.

It has been reported that aggression in individuals diagnosed with ASPD can be planned/proactive or impulsive/reactive [28]. Impulsivity is often seen together with aggression. It has been reported that this condition can be explained by low serotonergic activity [29]. Individuals with ASPD also frequently have comorbid diagnoses such as impulse control disorders, ADHD, and substance abuse. Along with these comorbid diagnoses, sudden/unplanned suicide attempts, a tendency to harm oneself and others during substance withdrawal, and aggression are common in individuals diagnosed with ASPD [30]. In our study, consistent with the literature, aggression, impulsivity, and suicide risk were found to be high among individuals diagnosed with ASPD.

In recent years, there has been an increase in the number of studies investigating the biological causes of behavioral patterns commonly seen in psychiatric disorders, such as impulsivity, aggression, criminal behavior, and suicide risk [31]. In our study, RBC levels were found to be low, and RDW and MCV levels were found to be high in individuals diagnosed with ASPD. There are a limited number of studies evaluating hematological parameters in individuals diagnosed with ASPD. In a study similar to ours, RDW levels were found to be high [29]. It has been reported that these differences in values may be related to alcohol and substance use, smoking, chronic stress factors, and nutritional irregularities [32]. Additionally, low RBC levels may also be caused by chronic stress affecting erythropoiesis [33].

Studies have associated low glucose levels with impulsivity and aggression [3]. This is consistent with the ASPD behavior pattern. Low glucose levels in individuals diagnosed with ASPD may be related to both irregular eating habits and alcohol and substance use. Impulsivity and self-harming behavior patterns have been associated with low total protein and albumin levels [34,35]. Recent studies on uric acid levels have increased in the field of psychiatry. It has been shown that uric acid levels are elevated in bipolar disorder and decreased in patients with MDD and anxiety disorder [36,37].

High uric acid levels are known to be associated with increased oxidative stress and metabolic syndrome. On the other hand, decreased hydration is known to reduce uric acid excretion [38]. In a study examining uric acid levels in ASPD, uric acid levels were found to be high, consistent with our study [39]. Elevated uric acid has been associated with impulsivity in many studies.

Low cholesterol levels have been associated with an increased risk of aggression and suicide in patients with psychiatric diagnoses [40,41]. Several clinical studies indicate sex-specific associations between lipid profiles and behavioral outcomes. For example, lower HDL cholesterol levels are significantly associated with increased aggression in women with schizophrenia, whereas analogous relationships in male cohorts are less consistently observed. Additionally, lipid modulation interventions have shown differential effects on aggression by sex, highlighting the need to consider sex differences when interpreting lipid–behavior relationships [42]. However, no studies have been found on the lipid profiles of individuals diagnosed with ASPD. In our study, no significant differences were found in values such as total cholesterol, LDL, and triglycerides. Only HDL levels were found to be lower in the patient group. The lack of significant differences in lipid profiles in our study may be related to methodological factors, such as the relatively limited sample size and the fact that the participant group consisted only of male individuals.

Studies on thyroid hormones have shown that hypothyroidism is associated with depressive symptoms, while hyperthyroidism is associated with anxiety and agitation [43]. In our study, however, no significant difference related to thyroid hormones were detected.

CRP, NLR, PLR, CAR, NAR, RDW, MPV, MCV, and SII are among the many parameters that can be measured through routine blood tests. They are markers of systemic inflammation and have been studied in many psychiatric disorders [44,45]. However, there are only a limited number of studies examining the relationship between personality disorders and inflammatory factors. A study conducted on patients with ASPD found a significant relationship between inflammatory factors and aggression and impulsivity [46]. Another study conducted on individuals with ASPD reported increased inflammatory factors and decreased BDNF [47]. In a study comparing schizophrenic patients who committed murder with schizophrenic patients who had no history of criminal behavior, CRP and CAR ratios were found to be high, while albumin levels were found to be low, consistent with our study. The authors considered inflammation as a potential biological counterpart to the pattern of lethal violent behavior, such as murder [48]. In our study, CRP and CAR values were also found to be relatively higher in murderers than in other groups of offenders.

Inflammatory factors such as interleukin 6, CRP, and tumor necrosis factor alpha have been reported to be elevated in patients who attempt suicide [49]. In a study investigating suicide risk in patients with schizophrenia, it was reported that inflammatory factors such as CAR, NAR, and CRP cannot be predictors of suicide risk but may determine the degree of suicide risk [50]. It has been reported that low cholesterol levels may affect serotonin levels, leading to an increase in depressive symptoms and thus a direct link to an increased risk of suicide [51]. No studies have been found examining the relationship between AST and ALT levels and suicide risk. Our study found a low level of positive correlation between ALT and suicide risk. ALT blood levels are considered normal between 10 and 40 U/L. Although the values in the ASPD group were lower than in the control group in our study, they were within normal limits in both groups. In the forensic ASPD group, despite higher rates of alcohol and substance use, the observed lower ALT levels may reflect the influence of several factors, including nutritional status, chronic substance exposure, drug use, and differences in muscle mass. The small sample size in our study may also have influenced these results. These factors may complicate the simple relationships between ALT and psychiatric diagnoses. In particular, while ALT shows a positive correlation with the likelihood of suicide, the fact that it does not predict ASPD itself highlights the complex and multifactorial nature of these biochemical–behavioral relationships.

One of the key findings of this study is that marital status, education level, serum uric acid, and the C-reactive protein/albumin ratio (CAR) were independently associated with antisocial personality disorder in multivariable analysis. However, these associations should be interpreted with caution. Given the cross-sectional design and the complex behavioral and environmental determinants of ASPD, the observed biological alterations cannot be considered etiological determinants. Rather, they may reflect downstream correlates of chronic stress exposure, substance use, nutritional factors, or social adversity commonly observed in forensic populations. Accordingly, these biomarkers should be viewed as potential correlates or risk indicators rather than causal mechanisms underlying ASPD.

### Limitations

The most significant limitation of the study is its cross-sectional design. The fact that only male individuals participated and the relatively small number of participants can be considered the second-most important limitation. Another limitation may be the failure to evaluate confounding factors that could affect participants’ blood parameters, such as diet types, exercise habits, or smoking. Another limitation is that the participants’ childhood behavioral problems were not assessed and the PCL-R test was not performed. Another important limitation of this study is the high incidence of psychiatric comorbidities frequently observed in patients with antisocial personality disorder. Although our study did not include ASPD cases with major psychiatric disorders, subthreshold comorbidities of conditions such as ADHD and anxiety disorders can independently influence inflammatory and metabolic biomarkers and potentially confound the observed relationship.

## 5. Conclusions

This study demonstrated increased inflammatory parameters and differences in hematological and biochemical parameters in male ASPD patients who have committed crimes. Significant findings include the increased risk of ASPD associated with CRP and uric acid as independent variables, and the relatively higher CRP levels in individuals who have committed homicide. There are a limited number of articles examining the concept of criminal behavior and biological parameters in ASPD, and no studies in the literature have evaluated biochemical, hematological, and inflammatory factors together. Our results make a significant contribution to the literature regarding the potential role of biological factors accompanying aggression, impulsivity, suicidal risk, and criminal behavior. Integrating routine blood parameters with psychometric assessments can improve early diagnosis and individualized risk management in forensic psychiatry practice.

## Figures and Tables

**Table 1:** Main characteristics of the individuals.

	Total (*n* = 113)	Control (*n* = 56)	Patient (*n* = 57)	*p*
Gender				
Female, *n* (%)				
Male, *n* (%)	113 (100)	56 (100)	57 (100)	
Age, median (min–max)	33 (18–54)	33 (20–54)	33 (18–47)	0.908
Marital status				**<0.001**
Single or widowed, *n* (%)	59 (52.2)	19 (33.9)	40 (70.2)	
Married, *n* (%)	54 (47.8)	37 (66.1)	17 (29.8)	
Level of education				**<0.001**
Primary school, *n* (%)	18 (15.9)	0 (0) *	18 (31.6) *	
Secondary school, *n* (%)	23 (20.4)	7 (12.5) *	16 (28.1) *	
High school, *n* (%)	40 (35.4)	19 (33.9)	21 (36.8)	
University, *n* (%)	32 (28.3)	30 (53.6) *	2 (3.5) *	
Alcohol use				**<0.001**
No, *n* (%)	88 (77.9)	56 (100)	32 (56.1)	
Yes, *n* (%)	25 (22.1)	0 (0)	25 (43.9)	
Substance use				**<0.001**
No, *n* (%)	82 (72.6)	56 (100)	26 (45.6)	
Yes, *n* (%)	31 (27.4)	0 (0)	31 (54.4)	
Legal problems due to alcohol/substance use				**<0.001**
No, *n* (%)	82 (72.6)	56 (100)	26 (45.6)	
Yes, *n* (%)	31 (27.4)	0 (0)	31 (54.4)	
Imprisonment due to alcohol/substance abuse				**<0.001**
No, *n* (%)	88 (77.9)	56 (100)	32 (56.1)	
Yes, *n* (%)	25 (22.1)	0 (0)	25 (43.9)	
Previous Inpatient Treatment				**<0.001**
No, *n* (%)	81 (71.7)	56 (100)	25 (43.9)	
Yes, n(%)	32 (28.3)	0 (0)	32 (56.1)	
Addiction Treatment Center (ATC) hospitalization				**<0.001**
No, *n* (%)	97 (85.8)	56 (100)	41 (71.9)	
Yes, *n* (%)	16 (14.2)	0 (0)	16 (28.1)	
Additional medical illness				**<0.001**
No, *n* (%)	98 (86.7)	56 (100)	42 (73.7)	
Yes, *n* (%)	15 (13.3)	0 (0)	15 (26.3)	
Alcohol and substance abuse in the family				**<0.001**
No, *n* (%)	101 (89.4)	56 (100)	45 (78.9)	
Yes, *n* (%)	12 (10.6)	0 (0)	12 (21.1)	
Signs of self-harm				**<0.001**
No, *n* (%)	67 (59.3)	56 (100)	11 (19.3)	
Yes, *n* (%)	46 (40.7)	0 (0)	46 (80.7)	
Suicide attempt				**<0.001**
No, *n* (%)	79 (69.9)	56 (100)	23 (40.4)	
Yes, *n* (%)	34 (30.1)	0 (0)	34 (59.6)	
Tattoo				**<0.001**
No, *n* (%)	76 (67.3)	56 (100)	20 (35.1)	
Yes, *n* (%)	37 (32.7)	0 (0)	37 (64.9)	

* *p* values significantly different between groups in each row, Categorical variables were presented as *n* (%). Normally distributed variables were presented as mean ± standard deviation, while skewed distributions were presented as median (min–max). Min: minimum; max: maximum.

**Table 2:** Comparison of scale scores between controls and patients.

	Total (*n* = 113)	Control (*n* = 56)	Patient (*n* = 57)	*p*
The Barratt Impulsiveness Scale-11				
Attentional score, median (min–max)	26 (9–41)	31 (14–41)	19 (9–30)	**<0.001**
Motor score, median (min–max)	15 (7–40)	12 (7–17)	25 (10–40)	**<0.001**
Non-planning, median (min–max)	22 (8–44)	20 (8–26)	31 (14–44)	**<0.001**
Total score, median (min–max)	66 (29–108)	63 (29–78)	74 (39–108)	**<0.001**
The Buss–Perry Aggression Questionnaire				
Physical aggression, median (min–max)	9 (0–45)	3 (0–6)	25 (9–45)	**<0.001**
Verbal aggression, median (min–max)	6 (1–29)	3 (1–6)	17 (6–29)	**<0.001**
Anger, median (min–max)	8 (0–35)	3 (0–8)	22 (7–35)	**<0.001**
Hostility, median (min–max)	9 (0–40)	2 (0–5)	24 (9–40)	**<0.001**
Total score, median (min–max)	39 (3–141)	12 (3–22)	86 (39–141)	**<0.001**
Suicide Probability Scale				
Hopelessness, median (min–max)	19 (11–41)	15 (11–19)	30 (18–41)	**<0.001**
Suicide ideation, median (min–max)	12 (9–28)	9 (9–13)	21 (11–28)	**<0.001**
Negative self-evaluation, median (min–max)	19 (9–48)	17 (15–20)	22 (9–48)	**<0.001**
Hostility, median (min–max)	9 (7–26)	8 (7–9)	15 (7–26)	**<0.001**
Total score, median (min–max)	56 (45–119)	49 (45–56)	91 (54–119)	**<0.001**

Bold values denote statistical significance at the *p* < 0.005 level. Skewed distributions were presented as median (min–max).

**Table 3:** Correlations between scale scores and laboratory values in patients.

	The Barratt Impulsiveness Scale-11		The Buss–Perry Aggression Questionnaire		Suicide Probability Scale	
	r	*p*	r	*p*	r	*p*
Age	−0.004	0.976	0.014	0.918	0.070	0.606
Height	0.117	0.388	0.222	0.097	0.206	0.124
Weight	0.024	0.860	0.096	0.479	0.208	0.121
WBC	−0.059	0.664	−0.003	0.983	−0.113	0.401
RBC	0.064	0.638	0.126	0.352	−0.024	0.862
HB	−0.041	0.765	0.149	0.269	0.065	0.631
PLT	−0.035	0.798	−0.115	0.394	−0.134	0.319
Neutrophil	0.022	0.868	0.062	0.645	−0.019	0.891
Lymphocyte	−0.134	0.321	−0.115	0.395	−0.206	0.125
Monocyte	−0.126	0.350	0.018	0.894	−0.110	0.417
Eosinophil	−0.221	0.099	−0.095	0.483	−0.247	0.064
Basophil	0.272	**0.041**	0.024	0.859	0.235	0.078
Glucose	0.034	0.803	0.038	0.778	−0.048	0.723
Urea	−0.175	0.193	−0.188	0.162	−0.111	0.410
Creatinine	−0.139	0.302	0.089	0.512	−0.139	0.303
Uric acid	−0.163	0.227	−0.167	0.214	0.002	0.986
Total protein	0.107	0.429	0.215	0.109	0.124	0.360
Albumin	0.213	0.111	0.220	0.101	0.130	0.335
AST	−0.238	0.074	−0.071	0.602	−0.023	0.867
ALT	−0.042	0.758	0.039	0.772	0.279	**0.036**
Cholesterol	−0.198	0.139	−0.105	0.437	0.040	0.765
HDL	−0.141	0.296	0.134	0.319	−0.162	0.229
LDL	−0.091	0.501	−0.117	0.388	0.136	0.313
Triglycerides	−0.055	0.683	−0.026	0.847	0.002	0.985
Na	−0.096	0.478	−0.010	0.942	0.013	0.924
K	−0.258	0.052	−0.102	0.451	−0.096	0.476
TSH	0.204	0.128	0.022	0.870	0.222	0.097
T3	−0.160	0.233	0.009	0.948	−0.017	0.903
T4	−0.120	0.374	0.065	0.631	0.009	0.949
B12	0.103	0.444	0.045	0.739	0.116	0.392
CRP	−0.149	0.268	−0.101	0.456	−0.087	0.518
CAR	−0.178	0.186	−0.135	0.315	−0.119	0.380
NAR	−0.023	0.865	−0.012	0.926	−0.023	0.864
NLR	0.129	0.341	0.108	0.423	0.136	0.311
MLR	−0.059	0.665	−0.012	0.928	0.031	0.821
PLR	0.119	0.380	0.046	0.736	0.116	0.392
SII	0.115	0.396	0.076	0.574	0.098	0.470

Bold values denote statistical significance at the *p* < 0.005 level.

**Table 4:** Laboratory parameters and inflammatory indices of the individuals.

	Total (*n* = 113)	Control (*n* = 56)	Patient (*n* = 57)	*p*
WBC ± SD	7.92 ± 1.99	7.81 ± 1.70	8.03 ± 2.25	0.555
RBC ± SD	5.23 ± 0.43	5.37 ± 0.34	5.09 ± 0.47	**<0.001**
Hb, median (min–max)	15.7 (10.2–17.8)	15.9 (13.4–17.4)	15.4 (10.2–17.8)	0.082
HCT ± SD	4.4 ± 2.9	45.8 ± 2	45.1 ± 3.5	0.189
MCV, median (min–max)	87.7 (60.4–103.1)	85.2 (77.3–90.6)	89.2 (60.4–103.1)	**<0.001**
PLT ± SD	250,550 ± 68,140	255,180 ± 64,518	246,000 ± 71,800	0.477
MPV ± SD	8.5 ± 0.9	8.5 ± 1.1	8.4 ± 0.9	0.704
Neutrophil, median (min–max)	4700 (1850–10,680)	4730 (2470–9020)	4700 (1850–10,680)	0.323
Lymphocyte, median (min–max)	2240 (260–4560)	2330 (990–4560)	2030 (260–3830)	0.065
Monocyte, median (min–max)	580 (200–1570)	580 (340–1570)	600 (200–1000)	0.836
Eosinophil, median (min–max)	140 (0–500)	150 (0–420)	120 (0–500)	0.143
Basophil, median (min–max)	40 (0–520)	40 (0–90)	5 (0–520)	0.143
RDW, median (min–max)	41.1 (34.7–54.3)	39.4 (34.7–48.1)	42.7 (35.4–54.3)	**<0.001**
Glucose, median (min–max)	86 (54–189)	89 (54–110)	81 (56–189)	**<0.001**
Urea, median (min–max)	26.4 (12–70)	29.5 (19–70)	25 (12–49)	**<0.001**
Creatinine ± SD	0.82 ± 0.12	0.83 ± 0.13	0.82 ± 0.12	0.650
Uric acid, median (min–max)	4.98 (0.81–8.58)	3.95 (0.81–7.86)	5.11 (3–8.58)	**0.019**
Total protein, median (min–max)	73 (55–82)	75 (67–80)	69 (55–82)	**<0.001**
Albumin, median (min–max)	44 (31–54)	46 (31–50)	43 (34–54)	**<0.001**
AST, median (min–max)	21 (12–84)	22 (13.7–84)	20 (12–49)	0.257
ALT, median (min–max)	21(7–121)	25 (7–121)	17 (8–61)	**0.001**
Cholesterol, median (min–max)	167 (98–343)	176 (105–307)	156 (98–343)	0.086
HDL, median (min–max)	41 (23–89)	45 (28–67)	40 (23–89)	**0.013**
LDL, median (min–max)	98 (38–257)	102 (58–209)	91 (38–257)	0.132
Triglycerides, median (min–max)	119 (11–386)	118 (50–386)	119 (11–345)	0.667
Na, median (min–max)	140 (133–147)	139 (135–147)	140 (133–144)	0.991
K ± SD	4.29 ± 0.29	4.34 ± 0.25	4.24 ± 0.31	0.062
TSH, median (min–max)	1.52 (0.28–4.07)	1.58 (0.28–3.12)	1.33 (0.28–4.07)	0.394
T3 ± SD	3.45 ± 0.4	3.42 ± 0.35	3.47 ± 0.45	0.530
T4 ± SD	0.87 ± 0.15	0.9 ± 0.14	0.84 ± 0.15	0.061
B12, median (min–max)	138 (50–639)	238 (101–402)	104 (50–639)	**<0.001**
CRP, median (min–max)	2.72 (0.96–29.3)	2.41 (0.96–5.45)	2.81 (1–29.3)	**0.031**
CAR, median (min–max)	0.06 (0.02–0.76)	0.05 (0.02–0.14)	0.07 (0.02–0.76)	**0.005**
NAR, median (min–max)	0.11 (0.04–0.28)	0.10 (0.05–0.23)	0.11 (0.04–0.28)	0.058
NLR, median (min–max)	2.13 (0.65–9.44)	1.87 (0.68–7.1)	2.42 (0.65–9.44)	**0.019**
MLR, median (min–max)	0.25 (0.14–3.42)	0.25 (0.16–1.01)	0.26 (0.14–3.42)	0.407
PLR, median (min–max)	116.7 (51.9–446.2)	107.7 (53.7–335.4)	123.8 (51.9–446.2)	0.118
SII, median (min–max)	491.4 (96.6–2771.8)	466.7 (145.4–2360.9)	538.4 (96.6–2771.8)	0.118

Bold values denote statistical significance at the *p* < 0.005 level. Categorical variables were presented as *n* (%). Normally distributed variables were presented as mean ± standard deviation, whereas skewed distributions were presented as median (min–max).

**Table 5:** Comparison of scales and inflammation indices among individuals with different types of criminality status.

	Using and/or Selling Substance (*n* = 10)	Murder (*n* = 5)	Theft (*n* = 11)	Involvement in Multiple Crimes (*n* = 18)	Other Crime (Sexual Abuse, Property Damage, Escape) (*n* = 13)	*p*
The Barratt Impulsiveness Scale-11, median (min–max)	70 (41–98)	70 (61–90)	75 (53–108)	73 (43–99)	75 (39–100)	0.974
The Buss–Perry Aggression Questionnaire, median (min–max)	81 (39–134)	88 (65–130)	106 (51–138)	82 (45–131)	86 (41–141)	0.244
Suicide Probability Scale, median (min–max)	80 (56–112)	94 (79–113)	92 (72–107)	91 (54–119)	91 (60–111)	0.662
CRP, median (min–max)	2.81 (1–13.8)	5.34 (2.25–17.6)	2.25 (1.48–28)	2.83 (1.24–29.3)	3.49 (1–11.2)	0.499
CAR, median (min–max)	0.07 (0.02–0.32)	0.13 (0.05–0.46)	0.05 (0.04–0.76)	0.06 (0.03–0.64)	0.08 (0.02–0.32)	0.534
NAR, median (min–max)	0.1 (0.05–0.21)	0.09 (0.05–0.28)	0.11 (0.06–0.2)	0.11 (0.04–0.2)	0.08 (0.02–0.32)	0.250
NLR, median (min–max)	2.19 (0.65–4.83)	2.42 (2–8.35)	1.89 (0.96–8.5)	2.36 (0.73–9.44)	2.47 (1.3–8.83)	0.587
MLR, median (min–max)	0.25 (0.16–0.56)	0.29 (0.2–3.42)	0.23 (0.14–0.88)	0.30 (0.18–0.71)	0.27 (0.17–1)	0.639
PLR, median (min–max)	110.1 (51.9–185.4)	134.3 (114.7–446.2)	116.7 (61.7–322.3)	114.7 (62.7–276.8)	150.8 (63.3–370)	0.623
SII, median (min–max)	544 (96.6–1541.8)	550 (402.9–1224.5)	491.4 (237.4–2771.8)	524.1 (116.1–2587.8)	795.8 (355.2–1961)	0.479

Categorical variables were presented as *n* (%). Normally distributed variables were presented as mean ± standard deviation, whereas skewed distributions were presented as median (min–max). Min: minimum; max: maximum.

**Table 6:** Multivariable logistic regression analysis of predictive factors of antisocial personality disorder.

	Unadjusted		Adjusted	
Risk Factors	OR (95% CI)	*p*	OR (95% CI)	*p*
Systemic immune-inflammation index (SII)	1.001 (1.0001–1.002)	**0.042**		
Uric acid	1.44 (1.13–1.84)	**0.003**	1.41 (1.04–1.93)	**0.028**
CRP	1.34 (1.09–1.65)	**0.005**	1.27 (1.02–1.58)	**0.033**
CRP albumin ratio (CAR)	3.96 (1.53–10.23)	**0.005**	3.36 (1.18–7.61)	**0.023**
Level of education (high school or university vs. primary or secondary)	0.1 (0.04–0.25)	**<0.001**	0.05 (0.01–0.18)	**<0.001**
Marital status (married vs. single or widowed)	0.22 (0.1–0.48)	**<0.001**	0.17 (0.06–0.52)	**0.002**

OR, odds ratio; 95% CI, 95% confidence interval. The *p* value of the Hosmere–Lemeshow test was 0.285. Bold values denote statistical significance at the *p* < 0.005 level.

## Data Availability

The data presented in this study are available on request from the corresponding author. The data are not publicly available because they contain information that could compromise the privacy of research participants.
